# Globalisation, rising authoritarianism, declining solidarity, and retreating multilateralism: A perfect storm for amplifying the risk of outbreaks

**DOI:** 10.1371/journal.pgph.0006783

**Published:** 2026-06-26

**Authors:** Winfred Dotse-Gborgbortsi, Madhukar Pai

**Affiliations:** 1 Department of Geography, University College London, London, United Kingdom; 2 School of Population and Global Health, McGill University, Montreal, Quebec, Canada; 3 Department of Medicine Solna, Karolinska Institutet, Stockholm, Sweden; PLOS: Public Library of Science, UNITED STATES OF AMERICA

We are more globally connected than ever before, and our destinies, regardless of who we are and where we happen to live, are inextricably intertwined. Climate change, conflicts, and disease outbreaks are powerful reminders of this simple fact. The movement of people, goods and services across borders has delivered enormous economic and social benefits, but it also accelerates the spread of infectious diseases [1]. International organisations such as the World Health Organisation (WHO), transnational disease surveillance systems, and shared financing mechanisms were designed to manage those risks collectively, and across geographical borders. That architecture is now crumbling, and two concurrent outbreaks, the Hantavirus outbreak linked to the MV Hondius cruise ship and Bundibugyo Ebola in the Democratic Republic of Congo (DRC), illustrate what happens when globalisation’s risks persist while its safeguards, safety nets, and global solidarity erode.

## The MV Hondius outbreak: A failure of collective response

The MV Hondius departed Argentina, on 1 April 2026. Two passengers died during the voyage, and a third died shortly after disembarkation and was confirmed to have Hantavirus infection [2]. By mid-May 2026, former passengers had been hospitalised or quarantined across 12 countries. The rapid spread across jurisdictions was a foreseeable consequence of modern cruise travel, which moves passengers of multiple nationalities across continents within days and makes contact tracing after disembarkation exceptionally difficult.

While the rapid, transnational spread of the virus was a clear justification for international cooperation, what followed was shaped more by politics than by epidemiology. Cape Verde lacked the capacity to manage a safe evacuation, while the Canary Islands initially refused to allow the vessel to dock, citing risks to the local population. International coordination remained limited as individual countries focused primarily on repatriating their own citizens from a ship carrying passengers from about 23 countries. This was not a failure of globalisation itself, but of the solidarity required to collectively manage its consequences. The failure of solidarity we observed during COVID-19, as evidenced by vaccine inequities [3], was on display yet again.

## Ebola in DRC and Uganda: Weakened health systems

A similar story is developing in Central Africa, where the third-largest Ebola outbreak is causing havoc. The current Ebola outbreak in DRC involves the Bundibugyo strain, for which no licensed vaccine or treatment exists, and which carries a case fatality rate of up to 50%. The WHO declared the outbreak a Public Health Emergency of International Concern (PHEIC) in May 2026, even though transmission had likely been happening for weeks before this [4]. Cases have already been confirmed in Kampala, Uganda, among individuals travelling from the DRC. Disease control efforts remain impeded by conflict, population movement and severe gaps in healthcare infrastructure.

This outbreak is occurring against a backdrop of sustained disinvestment in the health systems that depend on early detection and rapid response. The withdrawal of USAID financing and also by other G7 nations cut billions of dollars from sub-Saharan Africa, weakening surveillance networks, laboratory capacity and the community health workforce needed to identify outbreaks before they spread internationally [5]. DRC, in particular, was greatly reliant on USAID, and its abrupt demise has been consequential.

Several governments have responded with genuine political commitment to domestic resource mobilisation, and the WHO and Africa CDC have launched a joint continental preparedness and response plan [6], with a goal of raising US$ 518 million to support African countries together with partners to prepare for, rapidly detect and respond to the outbreak.

The push toward greater regional leadership, self-determination and self-sufficiency will ultimately strengthen health system resilience [7]. But shifting power and building that capacity takes years, and outbreaks do not wait. The gap between where health systems currently stand and where they need to be is already being measured in preventable deaths.

## Are we learning lessons?

The Hantavirus and Ebola outbreaks and the steep cuts to development financing are not unrelated. Both reflect a retreat from multilateral commitment at a moment when global interdependence demands the exact opposite. The COVID-19 pandemic demonstrated, at enormous cost, that this retreat is not only ethically insufficient but self-defeating. Infectious diseases do not spare the populations of countries that disengage from collective responses, nor can outbreaks be contained by rich nations sealing their borders.

That lesson appears to be going unlearned. It is disappointing that countries like USA and Canada have already imposed travel bans on some Ebola-affected African nations, instead of hurrying to support them during this crisis. The Ebola PHEIC is a test of global health solidarity and whether post-COVID commitments to reforming global health architecture is working [8]. Commitments to stronger international cooperation are being strained by rising authoritarianism, weakening multilateralism, aid cuts, and growing misinformation [9]. To contain the Ebola outbreak, WHO Member States must uphold their obligations under the International Health Regulations, including timely cross-border notification and resource-sharing. The response to the Ebola outbreak will show whether the lessons of COVID-19 have truly endured.

## Solidarity in action: What we should be doing

The word “solidarity” is used frequently in global health. What exactly is solidarity? The Global Health Solidarity Project’s framework [10] describes it as actively standing with others, not just in words but through meaningful action and shared commitments. It recognises that health is interconnected across borders, differences, and inequalities, and is built on trust, mutual respect, and inclusive decision-making. By working together toward common health goals, solidarity seeks not only to improve health outcomes but also to address the structural inequalities and power imbalances that drive health inequities.

**Fig 1:**
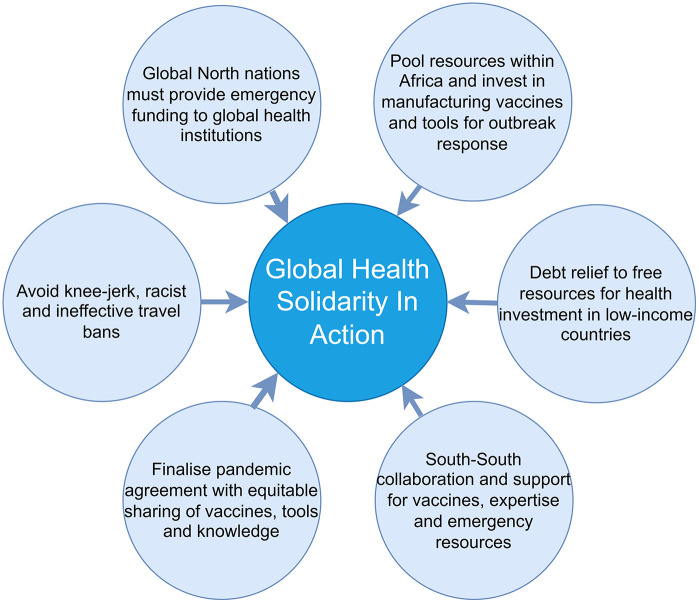
Examples of how global health solidarity could work during an outbreak.

As illustrated in Fig 1, global health solidarity in action should involve concrete actions, not just vague affirmations or declarations. For example, Global North nations must provide timely emergency funding to international agencies (such as WHO, Gavi, and CEPI) that respond to disease outbreaks and other global health emergencies. India’s support during the current Ebola crisis demonstrates the importance of South-South solidarity in strengthening emergency response efforts [11]. Within Africa, countries should pool resources and accelerate investment in regional infrastructure for vaccine manufacturing, medical supplies, and other outbreak-response logistics [12]. This is why the WHO and Africa CDC joint continental preparedness and response plan [6] is critical and should be fully funded. Strengthening regional capacity would improve preparedness and reduce dependence on external assistance during crises.

At the global level, all nations should work together to finalise the pandemic agreement [13], including provisions for equitable sharing of vaccines, therapeutics, knowledge, and other essential resources. Such measures would help ensure a more coordinated, effective, and fair response to future global health emergencies. Global North nations, in particular, must avoid using knee-jerk travel bans unless they are evidence-based, proportionate, and demonstrably effective in reducing disease transmission [14]. Greater emphasis should be placed on controlling outbreaks at their source before they spread locally and internationally.

The growing momentum toward African health sovereignty is both legitimate and necessary, but it should not be used to justify abrupt donor withdrawal and abandonment of solidarity and multilateralism by wealthy nations that have benefited from colonialism and imperialism. Regional self-sufficiency and international solidarity are not mutually exclusive. In an interconnected world, each depends on the other. Pathogens travel through the same global networks that economies depend upon. Weakening collective health systems does not reduce shared vulnerability; it amplifies it.
